# The First Record of *Echinococcus ortleppi* (G5) Tapeworms in Grey Wolf (*Canis lupus*)

**DOI:** 10.3390/pathogens10070853

**Published:** 2021-07-06

**Authors:** Jacek Karamon, Małgorzata Samorek-Pieróg, Jacek Sroka, Ewa Bilska-Zając, Joanna Dąbrowska, Maciej Kochanowski, Mirosław Różycki, Jolanta Zdybel, Tomasz Cencek

**Affiliations:** Department of Parasitology and Invasive Diseases, National Veterinary Research Institute, 24-100 Puławy, Poland; malgorzata.samorek-pierog@piwet.pulawy.pl (M.S.-P.); jacek.sroka@piwet.pulawy.pl (J.S.); ewa.bilska@piwet.pulawy.pl (E.B.-Z.); joanna.dabrowska@piwet.pulawy.pl (J.D.); maciej.kochanowski@piwet.pulawy.pl (M.K.); mrozycki@piwet.pulawy.pl (M.R.); j.zdybel@piwet.pulawy.pl (J.Z.); tcencek@piwet.pulawy.pl (T.C.)

**Keywords:** wolf, *Canis lupus*, *Echinococcus granulosus*, *Echinococcus ortleppi*, Poland

## Abstract

The aim of this study is to confirm the presence and molecular identification of *Echinococcus* tapeworms in wolves from south-eastern Poland. An investigation was carried out on the intestines of 13 wolves from south-eastern Poland. The small intestines were divided into three equal segments. Each segment was separately examined using the sedimentation and counting technique (SCT). The detected *Echinococcus* tapeworms were isolated and identified by PCRs and sequencing (*nad1* and *cox1* genes). Additionally, DNA isolated from the feces of wolves positive for *Echinococcus* tapeworms was examined with two diagnostic PCRs. The intestines of one wolf were positive for *E. granulosus s.l.* when assessed by SCT; the intestine was from a six-year-old male wolf killed in a communication accident. We detected 61 adult tapeworms: 42 in the anterior, 14 in the middle, and 5 in the posterior parts of the small intestine. The PCRs conducted for *cox1* and *nad1* produced specific products. A sequence comparison with the GenBank database showed similarity to the deposited *E. ortleppi* (G5) sequences. An analysis of the available phylogenetic sequences showed very little variation within the species of *E. ortleppi* (G5), and identity ranged from 99.10% to 100.00% in the case of *cox1* and from 99.04% to 100.00% in the case of *nad1*. One of the two diagnostic PCRs used and performed on the feces of *Echinococcus*-positive animals showed product specific for *E. granulosus*. This study showed the presence of adult *E. ortleppi* tapeworms in wolves for the first time.

## 1. Introduction

The genus *Echinococcus* consists of several species of tapeworms of zoonotic importance. The differences between the species concern the morphology of the parasites and their characteristic life cycles, mainly with a predilection to specific host species [1,2]. The genus includes, among others, *Echinococcus multilocularis*, which occurs in the northern hemisphere, where the red fox is the most common final host and rodents are the most common intermediate hosts. More locally-occurring species also exist and are related to local hosts. Among them are tapeworms that occur in South America, *E. vogeli* (hosts: bush dogs and pacas), *E. oligarthra* (hosts: American wild felids, agoutis, and pacas), and in the highlands of Tibet, *E. shiquicus* (hosts: Tibetan fox and plateau pika). In addition, the genus *Echinococcus* includes tapeworms grouped in the complex *E. granulosus sensu lato* (*s.l.*). This group of parasites includes several species additionally divided by genotype: *E. granulosus sensu stricto* (*s.s*.) (G1, G3), *E. equinus* (G4), *E. ortleppi* (G5), *E. canadensis* (G6–G8, G10), and *E. felidis*. *E. granulosus s.l.* is endemic worldwide but especially prevalent in areas where livestock breeding is practiced. In the domestic life cycle, the typical final host is the dog, and the intermediate hosts are domestic ungulates. However, in the sylvatic life cycle, wild canids, mainly wolves, are the most common final hosts (except *E. felidis*) and wild ungulates are the typical intermediate hosts [1,3].

The wolf (*Canis lupus*) is a predator inhabiting Eurasia and North America. Studies focused on *Echinococcus* spp. infection in wolves were conducted in various regions of the world. In North America, a high percentage of wolves infected with *E. granulosus s.l.* (62–63%) were reported [4,5]. Studies conducted in Asia also show the presence of *E. granulosus s.l.*, e.g., in a relatively high percentage in Kazakhstan (19.5%) [6], and lower in Mongolia (4.2%) [7]. In addition, *E. multilocularius* (3.4%) was also found in Mongolian wolves [7]. In Europe, cases of *E. granulosus s.l*. were also reported in wolves with a different percentage (3–26%) depending on the region [8,9,10,11,12,13]. Furthermore, 35.7% of wolves infected with *E. multilocularis* were found in a Slovakian study [14].

In recent years in Poland, the number of wolves has increased. Before wolves were put under protection, their range was limited to the Carpathians (south-eastern regions) and vast forests in the eastern and north-eastern parts of the country. The situation gradually started to change after wolves were recognized as a protected species (1998) [15]. Thanks to their ability to wander long distances, wolves return to their former refuges in central and western Poland, where they re-colonize all of the larger forest complexes [15,16]. In 2019, the population of wolves in Poland was estimated at 3222 individuals [17], of which about one-third were located in Podkarpackie Province (south-eastern part of the country).

In Poland, several studies have been carried out in wolf populations targeting intestinal parasites, mainly using microscopic methods [18,19], that have not been identified thus far. Moreover, interestingly, despite the examination of several hundred dogs, the presence of an *Echinococcus* infection in this species (considered the most common final host of this group of tapeworms) has not been found in Poland [20,21,22]. The only available reports on the detection of *E. granulosus* in definitive hosts in Poland come from studies conducted in the 1950s and 1960s, where authors found these tapeworms in foxes in fur farms [23,24]. One study confirmed that *E. granulosus*-positive foxes were fed with pig post-slaughter waste [23]. 

Some studies conducted around the world have attempted to identify the genotype/species of *E. granulosus s.l*. Most cases found *E. canadensis* [5,7,8,25,26,27] and one case found *E. granulosus s.s*. [28]. The presence of *E. ortleppi* in wolves has not been described thus far.

*E. ortleppi* was formerly known as the cattle strain (G5) of *E. granulosus* because cattle are considered the most common intermediate host [1,2]. Their larvae are most often found in the lungs of cattle, although cases of *E. ortleppi* (G5) cysts have also been confirmed in other host species: pigs, sheep, goats, buffaloes, camels [29,30,31,32], porcupines [33], and Philippine spotted deer [34]. Humans can also be an accidental intermediate host [35]; one case was confirmed in Poland [36]. The dog is considered the most common final host [2]. However, the presence of adult worms of this parasite in wild canids has not been confirmed so far.

The aim of this study is to confirm the presence and molecular identification of *Echinococcus* tapeworms in wolves from south-eastern Poland.

## 2. Results

**Figure 1:**
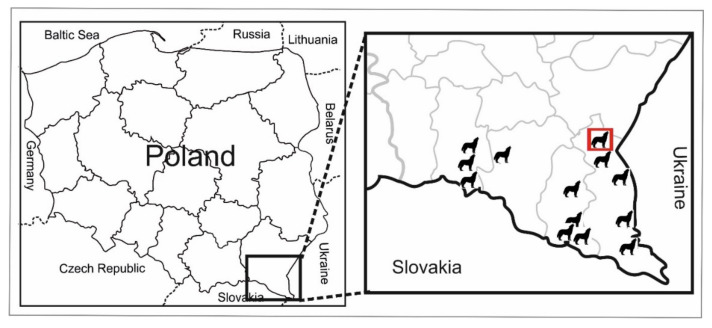
Location of wolves included in the study (the black icons represent wolves). Red square indicates the location where a wolf was positive for *Echinococcus ortleppi* was found.

**Figure 2:**
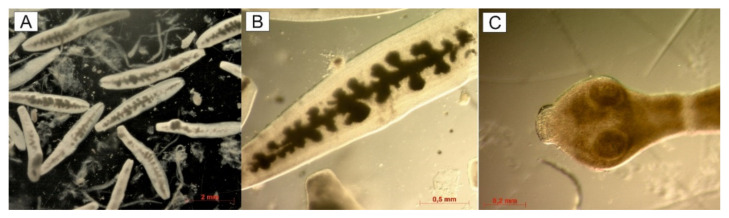
*Echinococcus ortleppi* adult tapeworms isolated from the small intestine of a wolf. (**A**) Free gravid proglottids. (**B**) Gravid proglottid with lateral sacculations of uterus. (**C**) Scolex.

Using the SCT method, the intestine of one wolf was found to be positive for *E. granulosus s.l*. The intestines were from a six-year-old male wolf killed in a communication accident approximately 15 km from the Ukrainian border (Figure 1). We detected 61 adult tapeworms (4–6 mm long): 42 in the anterior, 14 in the middle, and 5 in the posterior parts of the small intestine. Some free proglottids were also detected. Despite the worms being generally unpreserved and damaged, the characteristic shape of a uterus with lateral sacculations was observed in some gravid proglottids (Figure 2).

**Figure 3:**
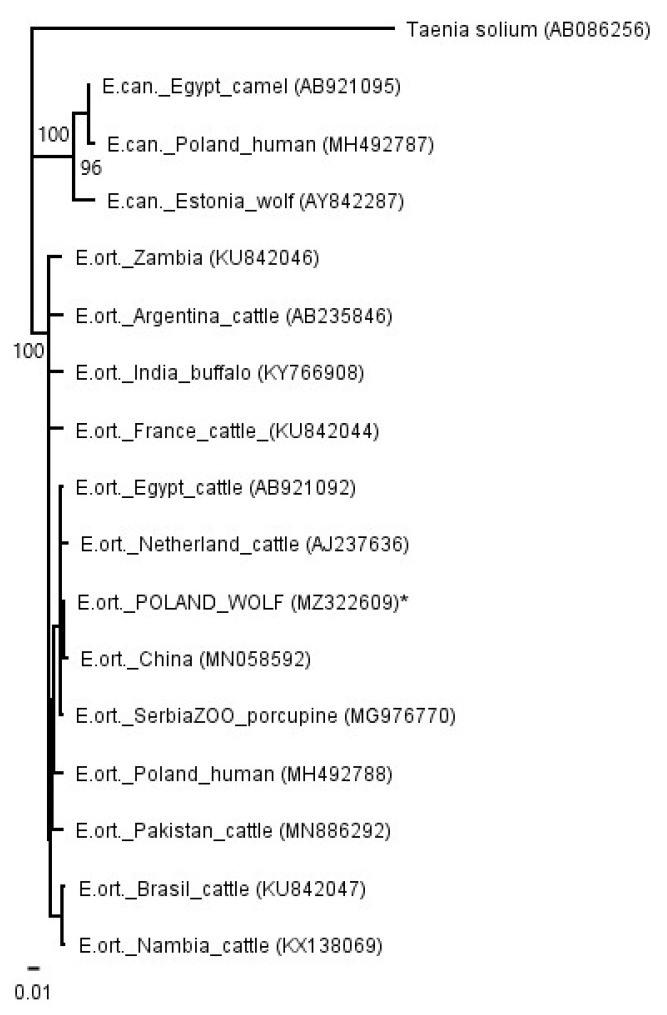
A phylogenetic tree based on a fragment of the nad1 gene. E. ort.—*Echinococcus ortleppi*; E. can.—*Echinococcus canadensis*. *—isolate from this study. The values on the tree nodes are bootstrap proportions (%).

**Figure 4:**
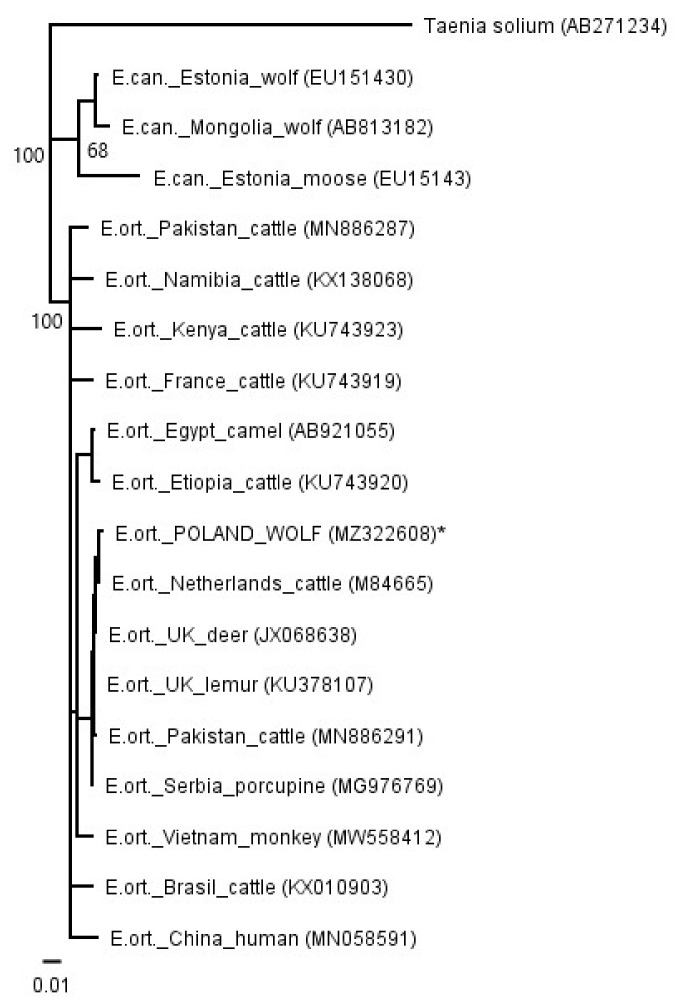
A phylogenetic tree based on a fragment of the cox1 gene. E. ort.—*Echinococcus ortleppi*; E. can.—*Echinococcus canadensis*. *—isolate from this study. The values on the tree nodes are bootstrap proportions (%).

The PCRs for *cox1* and *nad1* showed specific products. A sequence comparison with the GenBank database showed similarity to the *E. ortleppi* (G5) sequences deposited for both the *cox1* and *nad1* sequences (Figure 3 and Figure 4). An phylogenetic analysis of the available sequences showed very little variation within the species of *E. ortleppi* (G5); the identity ranged from 99.10% to 100.00% in the case of *cox1* and from 99.04% to 100.00% in the case of *nad1*. Full (100%) identity was observed in the *nad1* sequence isolated from the wolf in relation to the sequence previously isolated from the larvae from human in Poland [36], and, moreover, to *E. ortleppi* from Serbian zoo porcupines [33] and camels from Egypt [32]. In the analyzed *cox1* gene fragment, 100% identity occurred in comparison to isolates obtained from *E. ortleppi* larvae from Dutch cattle [37] and Philippine spotted deer (*Rusa alfredi*) from a zoo in the United Kingdom [34]. Relatively high similarities between all isolates analyzed with exemplary haplotypes of *E. canadensis* were also observed (93.02–93.43%).

Multiplex PCR [38] performed on the feces of *Echinococcus*-positive animal showed product specific for *E. granulosus*. A comparison of the sequence of this product with those in the GenBank database identified this sample as *E. ortleppi* (G5). However, the PCR, according to Abassi et al. (2003) [39], was negative.

## 3. Discussion

According to our knowledge, this is the first confirmed case of the detection of adult forms of *E. ortleppi* in wolves (*Canis lupus*). Until now, among the species belonging to the *E. granulosus s.l*. complex, only E*. canadensis* and *E. granulosus s.s*. were identified in wolves. In Estonia, *E. canadensis* (G10) was found in 4% of tested wolves; the genotype was confirmed by analyzing the *nad1* gene [8]. In Mongolia [7], tapeworms were found in five wolves (4.2%), which, after molecular identification, turned out to belong to the species *E. canadensis* (G6/G7 and G10). Moreover, in these studies, the presence of *E. multilocularis* (3.4%) was also reported. On the other hand, in Bulgaria [28] *E. granulosus s.s*. (G1) was identified in the wolf, and this genotype was also detected in all livestock and jackals examined in this area. Identification at the species level in the group of *E. granulosus s.l.* has also been performed by morphometric studies. However, this method requires well-preserved tapeworms isolated from the gut, which are often not available. In studies conducted in Wyoming (USA) [5], based on morphology (e.g., position and shape of vitelline glands and ovary), two samples isolated from wolves were identified to the species level as *E. canadensis* (G8/G10). Some studies also reported *E. granulosus s.l*. in wolves but without species (or genotype) identification, described as *E. granulosus*. This was probably due to the use of routine microscopic methods without precise morphological analysis and without molecular confirmation. However, in older studies, this was associated with the former parasitological nomenclature in which all species/genotypes were categorized under one name: *E. granulosus*. Therefore, it cannot be excluded that some of those cases reported as positive were *E. ortleppi*. In Italy (the Apennine Mountains) [13] in 1987–1999, *E. granulosus* was diagnosed in 15 examined wolves. Moreover, in these studies, the prevalence was significantly positively influenced by the local prevalence of cystic echinococcosis (CE) in sheep. *E. granulosus* was reported in wolves in Belarus (11.5%) [11] and Finland (26%) [12]. Moreover, older studies conducted in Lithuania (1970s) found *E. granulosus* in 1 wolf out of 41 examined [9]. Outside Europe, the occurrence of these tapeworms was also recorded without more precise identification of the strain or species: *E. granulosus* was found in the USA (Idaho and Montana) in 62.6% [4] of wolves and in Kazakhstan in 19.5% of wolves [6].

The intensity of infection of the wolf in our study was 61 tapeworms. Compared to most studies on wolves with *E. granulosus s.l*. in which this parameter was taken into account, we obtained a relatively low intensity. For example, in a study in the USA [4], only about 30% of wolves positive for tapeworms were infected with less than 100 tapeworms per animal and more than half of those had more than 1000 worms. Similarly, in Kazakhstan [6] and Italy [5], the average intensities of infection were 6533 and 6975 tapeworms per animal, respectively. On the other hand, only one to three tapeworms per animal were registered in Belarus [11]. However, it is difficult to draw conclusions from these comparisons because the species/genotypes were not identified in the studies. In our study, most of the tapeworms were found in the anterior part of the small intestine; this is consistent with the description of *E. granulosus*, which, unlike *E. multilocularis*, is more predisposed to the anterior and middle part of the intestine [40,41,42].

Cattle are considered the most common intermediate host of *E. ortleppi* (hence, this species was formerly called the cattle strain of *E. granulosus*), and the larval forms are most often located in the lungs. Therefore, in the typical development cycle of this parasite, cattle act as an intermediate host, and dogs are the final host [1,2]. However, the case of *E. ortleppi* in a wolf, presented in our research, may also suggest the presence of *E. ortleppi* cysts in wild cervids in this area. Cervids are the main food for wolves in Poland, and in the south-eastern region of the country, wolves preferentially prey on red deer (*Cervus elaphus*) [43]. This assumption may have been confirmed by the detection of *E. ortleppi* in cervid species (Philippine spotted deer, *Rusa alfredi*) in a British zoo [34]. However, the fact that this wolf could have been infected by the ingestion of cattle or sheep tissue with *Echinococcus* cysts should also be taken into account. However, it must be stressed that in Poland domestic ungulates are only secondary food components of the wolf diet (on average, they represent 5.2% of the food biomass consumed by these predators) [43]. 

*E. ortleppi* is also a zoonotic threat; some cases of cystic echinococcosis (CE) have been reported in humans in Europe, the Americas (South and North), Africa, and Asia [44,45,46,47,48,49]. In Poland, one case of *E. ortleppi* in humans was also reported [36], and it was the only case of infection by this parasitic species in Poland. The incident occurred in a 38-year-old woman, and the factor that predisposed her to infection was possibly her own unfenced garden (with access for animals) located near the forest.

In Poland, wolves have already been tested for *Echinococcus* spp. in the south of the country (Tatra National Park) [50], but the presence of *E. granulosus s.l*. DNA has not been confirmed. However, it should be noted that the authors performed their own PCR method without presenting sufficient basic validation parameters (sensitivity and specificity) concerning *E. granulosus s.l.* species. Therefore, the presence of *E. ortleppi* or other *E. granulosus s.l*. species in that population cannot be excluded.

More extensive investigation is needed in the dog population, which is the typical definitive host for this species. Thus far, studies in dogs in Poland for *Echinococcus* tapeworms have not shown the presence of *E. granulosus s.l*., in general [20,21]. The presence of *E. multilocularis* was found in 1.5% of the animals tested. Probably, the problem in detecting *E. granulosus s.l*. is due to the relatively low sensitivity of the method by Abbasi et al. (2003) [39]. In the present study, the presence of specific DNA in the stool of the wolf infected with *E. ortleppi* (SCT positive) was detected using the Multiplex PCR method [38]. However, the same fecal sample tested with the method mentioned above, according to Abbasi et al. (2003) [39], gave a negative result. This could suggest a lower sensitivity of the latter method in the detection of *E. granulosus s.l*. in feces, but it is hard to conclude based on one sample. Therefore, recently, there is an interest in developing a universal method for detecting *E. granulosus s.l*. infections, e.g., a recently published study presenting a set of qPCR methods covering all species of the *E. granulosus* complex [51].

The Polish haplotype isolated from wolves had the greatest similarity to isolates obtained from European *E. ortleppi* cysts [33,34,36,37] and to isolates from livestock in Egypt and China [32,52]. Full compliance was obtained using the Polish human isolate [36], which may indicate a common origin for both isolates. However, an analysis of the available sequences showed only little variation within the *E. ortleppi* species. Moreover, the similarity between both phylogenetic trees (*nad1* and *cox1*) is probably related to the common inheritance of mitochondrial genes. The relatively high similarity between all *E. ortleppi* analyzed isolates with exemplary haplotypes of *E. canadensis* is noteworthy; the two species are considered sister species [3,53,54]. 

## 4. Materials and Methods

### 4.1. Wolves

An investigation was carried in the intestines of 13 wolves (*Canis lupus*) from south-eastern Poland (NUTS PL821) (Figure 1); they were officially collected and necropsied by regional veterinary officers. There were eight male and five female wolves, aged from 0.5 to more than 15 years. Six of them were officially shot under permit by the Polish General Director of Environmental Protection, three were killed in communication accidents, three were killed by other wolves, and one died naturally (cachexia and damaged limb).

### 4.2. Sedimentation and Counting Technique (SCT)

The study material consisted of the intestines sent to a laboratory. The samples were stored for two weeks at <−70 °C before examination for safety reasons (to inactivate potentially present *Echinococcus* eggs). First, each small intestine was divided into three equal segments (anterior—A, middle—M, and posterior—P). Each segment was prepared separately and examined using the sedimentation and counting technique (SCT) [55,56] to find *Echinococcus* tapeworms. The detected *Echinococcus* spp. tapeworms were isolated during the SCT procedure and preserved in 70% ethanol. Additionally, the feces from a distal part of the large intestine of a wolf positive for *Echinococcus* in SCT were collected and frozen for further molecular examination. 

### 4.3. PCR and Sequencing

Before DNA extraction, *Echinococcus* tapeworms (isolated previously from the intestine) were washed in physiological saline in a petri dish. In this manner, three tapeworms were prepared and used for analysis. DNA was extracted from isolated *Echinococcus* tapeworms using a QIAamp DNA Mini Kit (Qiagen, Hilden, Germany), according to the manufacturer’s protocol. The fragments of two mitochondrial genes were amplified for analysis: *NADH dehydrogenase subunit 1* (*nad1*) and *cytochrom c oxidase* (*cox1*) subunit 1. PCR was performed according to the procedure by Bowles and McManus (1993) [37] using the primers JB11 (5′-AGATTCGTAAGGGGCCTAATA-3′) and JB12 (5′-ACCACTAACTAATTCACTTTC-3′) for *nad1* amplification. *Cox1* was amplified with PCR according to Casuli et al. (2008) [54] with the following primers: COI1 (5′-TTTTTTGGCCATCCTGAGGTTTAT-3′) and COI2 (5′-TAACGACATAACATAATGAAAATG-3′).

Additionally, DNA from samples of feces (from an *E. granulosus*-positive wolf) was extracted using the QIAamp DNA Mini Kit (Qiagen, Hilden, Germany), according to the manufacturer’s protocol for larger volumes of stool. The DNA samples were examined using multiplex PCR for the detection of *E. multilocularis*, *E. granulosus*, and other cestodes, including *Taenia* spp. [38] and by PCR for the detection of *E. granulosus* s.l. [39].

The amplicons obtained were separated by horizontal electrophoresis in a 1.5% agarose gel stained by Simply Safe (EURx, Gdańsk, Poland). The selected PCR products were sequenced by standard Sanger sequencing at a commercial company (Genomed S.A., Warsaw, Poland). The sequences obtained were compared to the GenBank collection using BLAST searches.

For phylogenetic analyses (*cox1* and *nad1*), sequenced fragments of *cox1* and *nad1* were edited and analyzed in Geneious R11 [57]. Previously trimmed sequences were aligned according to ClustalW using the following parameters: gap-opening penalty 10 and gap-extension penalty 0.2. For the phylogenetic trees, a Tamura–Nei genetic distance model and the neighbor-joining method were used in Geneious R11 [57]. One thousand nonparametric bootstrap inferences were performed. The nucleotide sequence data reported in this paper are available in the GenBank™ database under the following accession numbers: MZ322608—MZ322609. To estimate the phylogenetic position of the Polish isolate, homologous mitochondrial DNA sequences described earlier [8,29,32,33,34,36,37,52,58,59,60,61,62] were retrieved from GenBank and used in the analyses (GenBank accession numbers were presented in Figure 3 and Figure 4).

## 5. Conclusions

This study showed for the first time the presence of adult *E. ortleppi* in wolves (both visually and molecularly). Moreover, this is the first confirmed case in almost sixty years of *E. granulosus s.l*. in a definitive host in Poland and the first case of *E. ortleppi* in an animal host in this country. This indicates the need to continue research in this area with the use of sensitive methods in both wolf and dog populations, bearing in mind the particular zoonotic risk that CE caused by *E. ortleppi* in humans was confirmed in Poland a few years earlier [36].
